# From Valve to Vessel: Disseminated Staphylococcus aureus Endocarditis Leading to Splenic Artery Pseudoaneurysm and Mycotic Aneurysm

**DOI:** 10.7759/cureus.106700

**Published:** 2026-04-09

**Authors:** Hans Jesper F Del Mundo, Jillianne Unas, Adelaine Joy Espiritu, Manali Agrawal, Shailesh Aggarwal, Hardikkumar Bhanderi

**Affiliations:** 1 Internal Medicine, Monmouth Medical Center, Long Branch, USA

**Keywords:** infective endocarditis, intravenous drug user, mycotic aortic aneurysm, septic embolism, splenic artery pseudoaneurysm

## Abstract

Infective endocarditis (IE) is an infection of the endocardial surface of the heart, with *Staphylococcus aureus *as the leading cause in the United States. Identified risk factors include advanced age, male sex, intravenous drug use (IVDU), prior IE, structural or valvular heart disease, HIV infection, and chronic hemodialysis. In IVDU, IE most commonly affects the right-sided valves, while bivalvular involvement is uncommon but associated with more severe disease and increased risk of rapid deterioration. Multiple complications arise from IE, including variable vascular phenomena and systemic embolic complications.

A 62-year-old male with a history of polysubstance abuse and intravenous drug use (heroin) presented with altered mental status. He was febrile and tachycardic, with laboratory studies revealing a white blood cell count (WBC) of 24.8 × 10³/µL, an elevated erythrocyte sedimentation rate (ESR) of 130 mm/hr, and a C-reactive protein (CRP) of 248 mg/L. Blood cultures grew methicillin-sensitive *Staphylococcus aureus *(MSSA). Transesophageal echocardiography revealed vegetations on both the mitral and tricuspid valves, confirming bivalvular IE. His disease course was complicated by a cardioembolic stroke, L1-L2 osteomyelitis, bilateral psoas abscesses, septic pulmonary emboli, and splenic infarctions. During hospitalization, he developed a ruptured splenic artery pseudoaneurysm (SAPA), successfully managed with selective endovascular embolization requiring multiple blood transfusions and ICU-level monitoring for hemodynamic instability. Following stabilization, he returned to the medical floor to continue intravenous antibiotics and supportive care, but subsequently developed an enlarging abdominal aortic mycotic aneurysm (MA). Patient underwent mitral and tricuspid valve replacements, which were complicated by second-degree atrioventricular block progressing to complete heart block, necessitating permanent pacemaker placement. His hospital course was further complicated by bilateral pleural effusions requiring thoracentesis and a pericardial effusion requiring emergent subxiphoid pericardial window creation. He was discharged to a rehabilitation facility on chronic suppressive therapy with oral cephalexin and levofloxacin for a concomitant *Serratia* infection and subsequently underwent endovascular aortic repair.

This case highlights the aggressive and disseminated course of *S. aureus *IE in IVDU, with bivalvular involvement, which may be associated with an increased risk of systemic embolization. Large, mobile vegetations exceeding 10 mm in size, especially when located on the left-sided valves, are strong indications for early surgical intervention. While the brain and spleen are common embolic sites, this case demonstrates rare vascular complications such as SAPA and MA, which can be life-threatening due to the risk of rupture and hemorrhage. The patient ultimately required bivalvular replacement, which also increases the risk of heart block due to the proximity of the valves to the conduction system. Early detection through imaging, timely multidisciplinary management, and consideration of surgical or endovascular intervention even during active infection are critical for improving outcomes in patients with complex, disseminated IE.

## Introduction

Infective endocarditis (IE) is an infection of the heart's endocardial surface, most commonly involving the cardiac valves. *Staphylococcus aureus *is the leading cause of IE in the United States. Approximately 30% of individuals are colonized with *S. aureus*, which can transition from a benign commensal to an invasive pathogen when host barriers are disrupted. This can result in bacteremia and progress to IE in susceptible individuals. Identified risk factors include advanced age, male sex, intravenous drug use (IVDU), prior IE, poor dentition, structural or valvular heart disease, congenital heart disease, HIV infection, and chronic hemodialysis, among others [1].

Despite its relatively low incidence at an estimated 30 cases per million annually and approximately 40,000 to 50,000 cases per year in the United States, IE remains a disease associated with significant morbidity and mortality, with reported hospital mortality rates of 15-25% within three months of diagnosis. IE-related hospitalizations in the United States have increased in recent years with a concomitant decline in in-hospital mortality, likely reflecting advances in diagnostic modalities, antimicrobial therapy, and surgical management. The ongoing opioid crisis in the United States, with at least one in ten Americans reporting illicit drug use, has further contributed to this rising trend, as IE continues to be one of the most serious complications associated with IVDU [2].

In patients with a history of IVDU, infective endocarditis most commonly affects the right-sided heart valves, particularly the tricuspid valve, in contrast to non-IVDU patients, where left-sided valves are more frequently involved [3]. Bi-valvular IE in patients with a history of IVDU is uncommon but clinically significant, which occurs in approximately 10-19% of cases [4]. Compared with single-valve involvement, the clinical course of bi-valvular IE appears to be more severe, with an increased risk of rapid clinical deterioration. Multiple complications arise from IE, including variable vascular phenomena and systemic embolic complications, which include septic embolism, splenic artery aneurysm (SAA) and pseudoaneurysm (SAPA), mycotic aneurysm (MA), and psoas abscess [5-8].

Septic embolism occurs when infected valvular vegetations fragment and disseminate through the bloodstream, leading to occlusion and infection of distal vessels. On the other hand, splenic artery aneurysm (SAA), which is a rare but recognized complication of IE, occurs in approximately 1-10% of the population. The increasing use of computed tomography angiography (CTA) and magnetic resonance imaging (MRI) has enhanced the detection of true SAAs and splenic artery pseudoaneurysms (SAPAs), up to 90% of which remain clinically silent or present with abdominal pain. Pseudoaneurysm formation typically arises secondary to septic embolization to distal arterial branches, resulting in vasa vasorum occlusion, vessel wall necrosis, and subsequent pseudoaneurysm development [9-11].

Mycotic aneurysm (MA) is a rare complication of infective endocarditis. It is a localized, abnormal dilatation of an arterial wall that arises when septic emboli lodge, especially in arterial branches or vessel bifurcations, leading to infection, destruction of the vessel wall, and subsequent aneurysm formation. The most common infection sites are the femoral artery and abdominal aorta, followed by the thoraco-abdominal aorta [12-13]. It poses a high morbidity and mortality, primarily if not addressed promptly.

Infective endocarditis can also be complicated by heart block in 4-5% of cases overall, with risk substantially increased in patients with invasive disease or paravalvular abscess [14-16]. In these cases, the incidence of conduction abnormalities can be as high as 53% and is associated with a worse prognosis [17].

Although uncommon, splenic artery pseudoaneurysms and mycotic aneurysms represent life-threatening complications of IE due to their high risk of rupture and potentially fatal intra-abdominal hemorrhage. Prompt recognition and timely intervention are, therefore, essential to prevent catastrophic outcomes. We present a case of methicillin-sensitive *Staphylococcus aureus* (MSSA) bacteremia secondary to infective endocarditis in the setting of injection drug use, complicated by stroke, osteomyelitis, bilateral psoas abscesses, splenic artery pseudoaneurysm, mycotic aneurysm, and heart block.

## Case presentation

**Figure 1:**
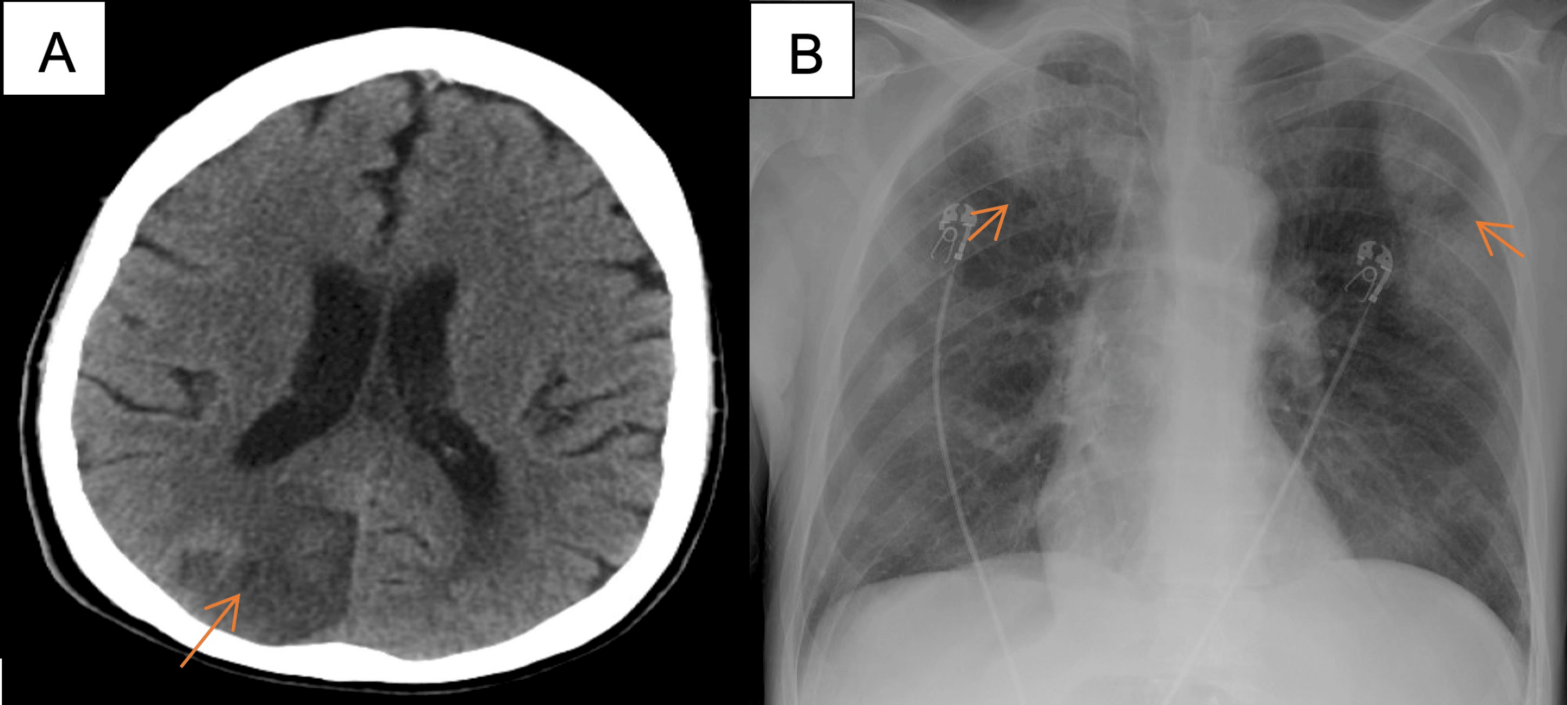
Non-contrast CT head and chest X-ray (A) Non-contrast CT head showing a recent-appearing right parieto-occipital infarct; (B) chest X-ray showing multifocal hazy opacities.

The patient is a 62-year-old male with a past medical history of polysubstance abuse and IVDU who was brought to the emergency department after being found unconscious in a garage by a friend. He reported daily heroin use. On arrival, he was febrile (102°F) and tachycardic. Initial laboratory studies showed white blood cell count of 24.8 ×10³/µL (4-10 ×10³/µL), elevated erythrocyte sedimentation rate (ESR) of 130 mm/hr (0-20 mm/hr), and C-Reactive Protein (CRP) of 248 mg/L (<=7 mg/L). Urine toxicology was positive for opiates, norfentanyl, and cocaine. CT head demonstrated a recent-appearing right parieto-occipital infarct (Figure 1A). The patient was empirically started on piperacillin-tazobactam and vancomycin for sepsis from pneumonia, as the chest radiograph showed multifocal hazy opacities concerning for pneumonia (Figure 1B). However, infective endocarditis was then considered when blood cultures grew *Staphylococcus aureus*. Antibiotics were subsequently narrowed to intravenous cefazolin based on susceptibility. Further infectious workup revealed negative HIV serology but positive hepatitis C antibody with hepatitis C virus (HCV) RNA viral load of 198,000 IU/mL.

**Figure 2:**
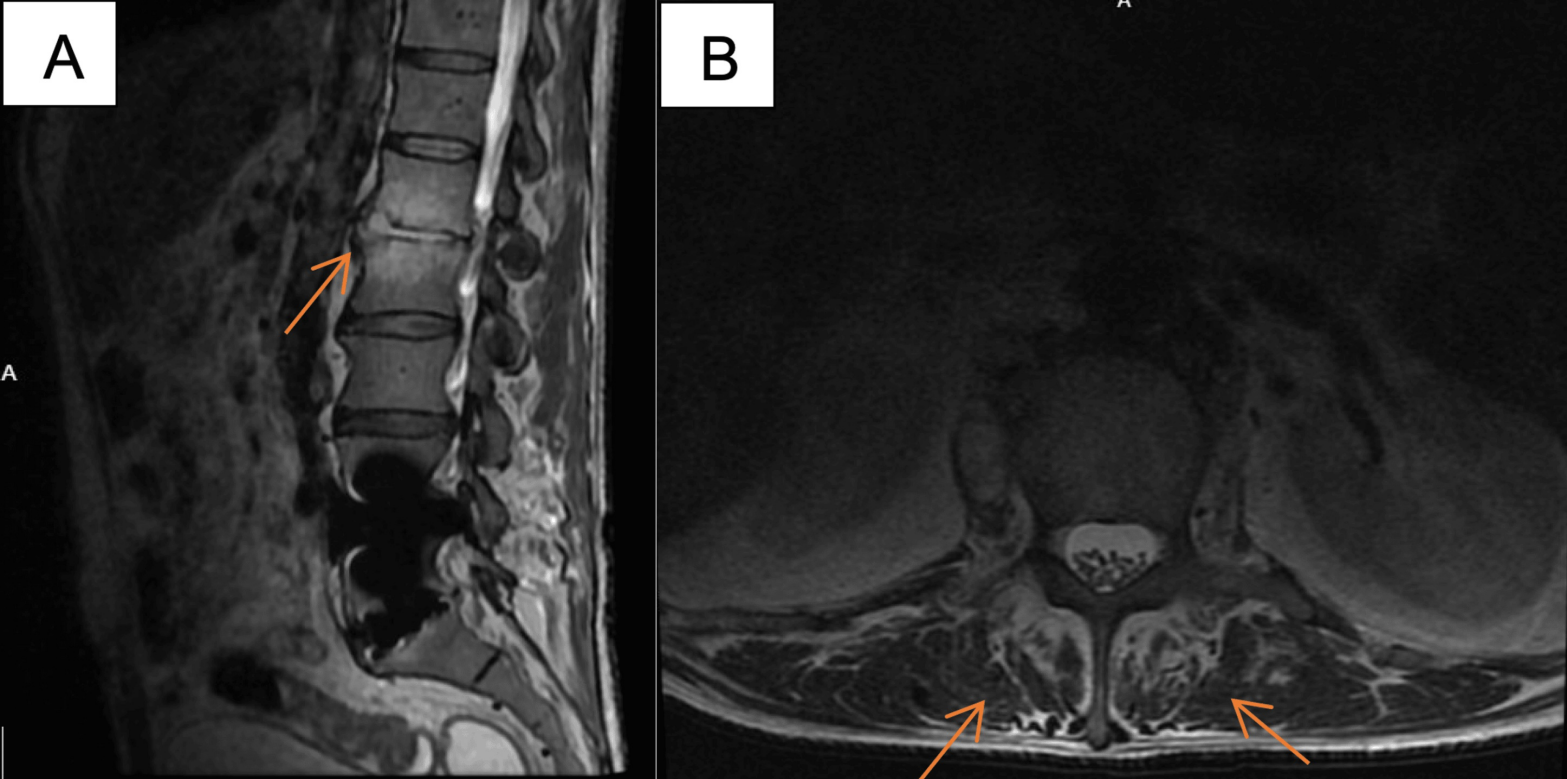
MRI lumbar spine T2 (A) L1-L2 osteomyelitis on sagittal view; (B) bilateral psoas abscesses on axial view.

During admission, he developed severe back pain. MRI of the lumbar spine revealed acute L1-L2 osteomyelitis/discitis without epidural abscess (Figure 2A), but with adjacent bilateral psoas abscesses (Figure 2B). Persistent tachypnea and tachycardia prompted CTA of the chest, which demonstrated multiple small peripheral cavitary pulmonary lesions consistent with septic emboli, along with splenomegaly and anterior splenic infarcts.

**Figure 3:**
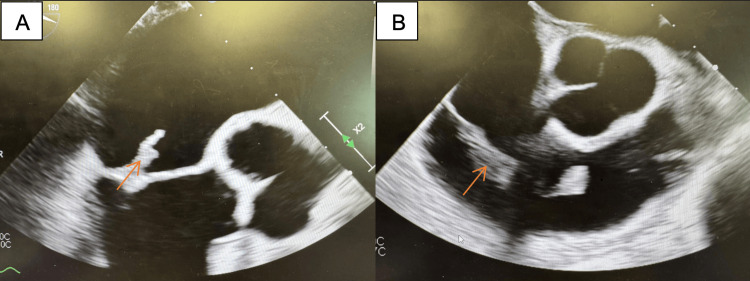
Transesophageal echocardiogram images (A) Mitral valve vegetation (1.6-1.7 cm); (B) tricuspid valve vegetation (4.5 cm).

Transesophageal echocardiography (TEE) revealed a large mitral valve vegetation measuring 1.6-1.7 cm (Figure 3A) and a very large mobile vegetation approximately 4.5 cm on the ventricular surface of the tricuspid valve (Figure 3B). At this point, no evidence of heart block or prolonged PR interval was noted on ECG and telemetry. The patient was initially transferred to a tertiary center for surgical evaluation but was later returned to our institution for continued management, as he was deemed a poor operative candidate primarily due to the high risk of recurrent infection in the setting of active intravenous drug use. 

**Figure 4:**
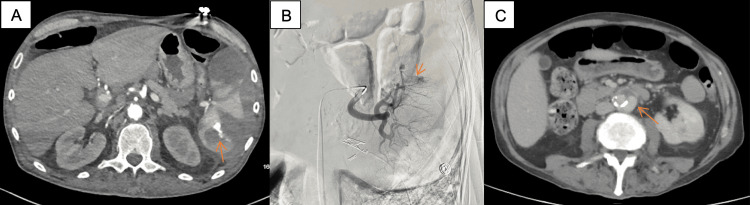
CT angiography chest abdomen and pelvis (A) Splenic artery pseudoaneurysm; (B) splenic artery angiography with third-order embolization of two inferior pole branches supplying a pseudoaneurysm and an area of active extravasation; (C) CT abdomen and pelvis with IV contrast showing abdominal aortic mycotic aneurysm.

Drainage of the psoas abscesses was requested, but repeat CT abdomen revealed a rim-enhancing left psoas collection, an ill-defined right psoas collection, and a 3.2 × 2.2 × 1.9 cm hyperenhancing splenic lesion concerning for pseudoaneurysm (Figure 4A). Shortly thereafter, the patient developed diffuse abdominal tenderness and an acute drop in hemoglobin from 7.6 g/dL to 4.8 g/dL. CTA confirmed worsening splenic infarcts, extensive subcapsular splenic hematoma, and moderate hemorrhagic ascites. Due to prohibitive operative risk, General Surgery deferred definitive splenectomy. Interventional Radiology was consulted and performed splenic artery angiography with third-order embolization of two inferior pole branches supplying the pseudoaneurysm and an area of active extravasation (Figure 4B). The patient required multiple blood transfusions and ICU-level monitoring for hemodynamic instability. After stabilization, he was transferred back to the medical floor to finish a planned 42-day course of intravenous cefazolin initiated from the date of a negative blood culture; however, during the last week of therapy, the patient developed another episode of severe abdominal pain, which prompted repeat imaging. CTA showed an increasing abdominal aortic aneurysm (AAA)measuring 3.5 x 3 cm, with two penetrating ulcers (the larger measuring 14 mm) (Figure 4C). The patient was deemed unfit for acute vascular intervention, given a history of bacteremia and septic emboli in the setting of IVDU. Repeat blood cultures sent to rule out ongoing bacteremia were negative. 

The patient was symptomatically managed and prepared for transfer to a tertiary center for anticipated vascular intervention. After transfer, the patient was evaluated by cardiothoracic surgery for reevaluation of the mitral and tricuspid valves before proceeding with AAA repair. Repeat TEE showed mitral valve vegetation with mild regurgitation and tricuspid valve vegetation with moderate regurgitation. The patient underwent mitral valve replacement and tricuspid valve replacement with placement of mediastinal and right chest tubes. Definitive mycotic aneurysm repair was deferred, given asymptomatic status, and the prohibitive risks associated with recent open heart surgery.

The hospital course was complicated by a second-degree heart block, possibly due to recent cardiac surgery, which progressed to complete heart block with narrow complex junctional escape, necessitating a permanent pacemaker (PPM) placement. Before this procedure, the patient developed fever and sternal pain, which was found to be due to bilateral pleural effusions for which he underwent thoracentesis. Intravenous cefazolin was switched to oral cefalexin. 

Following leadless PPM placement, pericardial effusion was noted on echocardiography, which required emergent subxiphoid pericardial window creation, during which 150 mL of serous fluid was evacuated. Fluid cultures were positive for *Serratia *species, prompting empiric treatment with ertapenem, which was later narrowed to levofloxacin. The patient’s condition stabilized, and he was discharged to a rehabilitation facility on suppressive oral cefalexin, concurrent with levofloxacin for the treatment of the *Serratia *infection, and subsequently underwent endovascular aortic repair.

## Discussion

This case illustrates the spectrum of disseminated *Staphylococcus aureus *infection in the context of intravenous drug use (IVDU)-associated infective endocarditis (IE). While patients with IVDU are predisposed to right-sided IE, our patient demonstrated bi-valvular involvement, which possibly increased the risk of systemic embolization. Bi-valvular involvement highlights the risk of swift progression to multifocal disease, evidenced by septic emboli, vertebral osteomyelitis, and bilateral psoas abscesses. Literature further supports higher rates of congestive heart failure, systemic embolic events (such as stroke), persistent bacteremia, and perivalvular complications [3-5]. The presence of large vegetations (>10 mm) has been strongly associated with an increased risk of systemic embolization, which is a significant determinant of adverse outcomes. The brain and spleen represent the most common sites of embolic events. Bi-valvular involvement is also associated with a more frequent need for early surgical intervention, and surgery is linked to improved survival in these cases [6,7]. 

A national inpatient sample from the US showed concurrent heart block in 867 (4.6%) among 18,733 patients admitted with infective endocarditis [16]. The severity of infective endocarditis, specifically the presence of invasive infection (such as an abscess or paravalvular regurgitation), is closely linked to ECG changes in cardiac conduction. Conduction abnormalities occurred in 53% of patients with invasive IE, compared to only 26% of those with isolated valve infection. Furthermore, patients with new or unknown duration intraventricular blocks faced a 41% mortality rate, significantly higher than the 15% rate for those without these blocks. These findings suggest that conduction abnormalities in IE are an indicator of advanced disease rather than simply a fatal arrhythmia [17]. 

Splenic complications occur in 20-40% of left-sided IE cases, most commonly as infarction or abscess formation. Splenic artery pseudoaneurysm (SAPA) is rare and potentially fatal if ruptured [9]. It may be clinically silent or present with abdominal pain, as in this case. It typically arises from septic emboli or direct vessel wall invasion, posing a risk for catastrophic intra-abdominal hemorrhage. Diagnosis relies on imaging, particularly CTA, and management often involves endovascular embolization or splenectomy. Specifically for this patient, surgical intervention was contraindicated due to comorbidities, and splenic artery embolization was lifesaving [18]. Endovascular treatment is safe and effective. Radiologic screening with abdominal CTA allows early detection of asymptomatic SAAs, which can then be managed to prevent rupture. Delayed formation of SAAs may justify repeat imaging after antibiotic therapy. In a Mayo Clinic series of 217 SAAs, only three cases (1.4%) were associated with IE. Similarly, Tessier et al. identified 37 visceral artery pseudoaneurysms, 10 (27%) of which involved the splenic artery [9,11]. Regardless, CTA should not be routinely performed unless it is clinically indicated, like in the case of this patient who presented with severe abdominal pain despite antibiotic treatment.

The American Association for Thoracic Surgery (AATS) guidelines recommend urgent or even emergency surgery in patients with left-sided native valve endocarditis (NVE) or prosthetic valve endocarditis (PVE) who exhibit mobile vegetations greater than 10 mm in length with clinical evidence of embolic phenomenon despite appropriate antibiotic therapy [19]. Large mobile vegetations greater than 10 mm on the anterior mitral valve leaflet are associated with a higher risk of embolization. Location, size, and mobility of vegetation, prior embolism, type of organism, and duration of antimicrobial therapy all influence the risk of a subsequent embolic event. The trend is to be more aggressive and operate on patients at imminent risk of embolism earlier. A recurrent embolic event may occur at any point and may be devastating [8].

Mycotic aneurysms (MA) account for approximately 0.7% to 4.5% of all aortic aneurysms, with the abdominal aorta identified as one of the most frequent sites of involvement. Although the suprarenal aorta is more commonly affected, several surgical series have demonstrated that a considerable proportion of abdominal mycotic aneurysms occur in the infrarenal segment, comprising 54% to 61% of reported cases [12, 13]. The imaging of choice for SAA is a contrast-enhanced CTA. Suggestive features include periaortic soft-tissue stranding, fluid, a concentric inflammatory response, or periaortic gas, all of which are suggestive of infection. Furthermore, the absence of mural calcification and rapid aneurysm enlargement further supports the diagnosis. Management of MA, in conjunction with the initiation of appropriate IV antibiotics, includes open surgical repair vs endovascular repair. Antimicrobial therapy alone is associated with high mortality and variable prognosis. Endovascular repair is preferred only for higher-risk patients as it carries a risk of persistent infection [20]. 

This case highlights the critical role of multidisciplinary care, including infectious disease, cardiology, interventional radiology, general surgery, and critical care, in the management of high-risk IE patients. Early recognition and prompt intervention are essential to prevent catastrophic outcomes, particularly in the setting of complications such as splenic artery pseudoaneurysm and aortic mycotic aneurysm.

## Conclusions

Disseminated *Staphylococcus aureus *infective endocarditis in patients with IVDU can lead to severe, life-threatening complications beyond cardiac involvement. This case highlights the rare but critical occurrence of splenic artery pseudoaneurysm and mycotic aneurysm, which may arise from septic embolization in the setting of IE. Early recognition through advanced imaging, timely intervention with endovascular embolization, and coordinated multidisciplinary management are essential for preventing catastrophic outcomes. For high-risk patients who are poor surgical candidates, minimally invasive endovascular approaches provide a lifesaving alternative to splenectomy. This case underscores the importance of vigilance for uncommon yet potentially fatal complications in IE and reinforces the value of individualized, guideline-informed care.
